# Differential regulation of the Rac1 GTPase–activating protein (GAP) BCR during oxygen/glucose deprivation in hippocampal and cortical neurons

**DOI:** 10.1074/jbc.M117.796292

**Published:** 2017-10-18

**Authors:** Katharine R. Smith, Dipen Rajgor, Jonathan G. Hanley

**Affiliations:** From the ‡ Centre for Synaptic Plasticity and School of Biochemistry, Biomedical Sciences Building, University of Bristol, University Walk, Bristol BS8 1TD, United Kingdom and; the § Department of Pharmacology, University of Colorado Denver School of Medicine, Aurora, Colorado 80045

**Keywords:** GTPase-activating protein (GAP), guanine nucleotide exchange factor (GEF), hippocampus, ischemia, NMDA receptor (NMDAR), BCR, cortex, OGD, Tiam1

## Abstract

Brain ischemia causes oxygen and glucose deprivation (OGD) in neurons, triggering a cascade of events leading to synaptic accumulation of glutamate. Excessive activation of glutamate receptors causes excitotoxicity and delayed cell death in vulnerable neurons. Following global cerebral ischemia, hippocampal CA1 pyramidal neurons are more vulnerable to injury than their cortical counterparts, but the mechanisms that underlie this difference are unclear. Signaling via Rho-family small GTPases, their upstream guanine nucleotide exchange factors, and GTPase-activating proteins (GAPs) is differentially dysregulated in response to OGD/ischemia in hippocampal and cortical neurons. Increased Rac1 activity caused by OGD/ischemia contributes to neuronal death in hippocampal neurons via diverse effects on NADPH oxidase activity and dendritic spine morphology. The Rac1 guanine nucleotide exchange factor Tiam1 mediates an OGD-induced increase in Rac1 activity in hippocampal neurons; however, the identity of an antagonistic GAP remains elusive. Here we show that the Rac1 GAP breakpoint cluster region (BCR) associates with NMDA receptors (NMDARs) along with Tiam1 and that this protein complex is more abundant in hippocampal compared with cortical neurons. Although total BCR is similar in the two neuronal types, BCR is more active in hippocampal compared with cortical neurons. OGD causes an NMDAR- and Ca^2+^-permeable AMPAR-dependent deactivation of BCR in hippocampal but not cortical neurons. BCR knockdown occludes OGD-induced Rac1 activation in hippocampal neurons. Furthermore, disrupting the Tiam1–NMDAR interaction with a fragment of Tiam1 blocks OGD-induced Tiam1 activation but has no effect on the deactivation of BCR. This work identifies BCR as a critical player in Rac1 regulation during OGD in hippocampal neurons.

## Introduction

Global cerebral ischemia causes widespread depolarization of the neuronal plasma membrane, release of the excitatory neurotransmitter glutamate, and overexcitation of ionotropic glutamate receptors, leading to sustained elevation of intracellular Ca^2+^ and, consequently, a delayed, selective cell death (1). Specific regions of the brain show greater neuronal injury than others, suggesting that different mechanisms are recruited in response to insult, leading to cell death. Pyramidal neurons in the hippocampal CA1 subregion are the most vulnerable, whereas CA3 pyramidal neurons are resistant. Although cortical pyramidal neurons are affected by ischemia, they are less vulnerable than those in hippocampal CA1 (2, 3). Moreover, dissociated cultures of hippocampal neurons are more vulnerable to oxygen/glucose deprivation (OGD)^3^ than equivalent cultures prepared from cerebral cortex, suggesting that distinct cell type–specific mechanisms are activated in response to insult (4).

Rho-family GTPases are proteins of fundamental importance in integrating intracellular signaling pathways. They function as molecular switches, cycling between an active GTP-bound state and an inactive GDP-bound state and, when activated, bind to a wide range of effectors to initiate a diverse array of signaling pathways that control numerous cell biological processes via effects on actin dynamics, such as cell migration, morphogenesis, and vesicle trafficking as well as gene transcription, cell cycle progression, and cell survival. The precise spatial and temporal regulation of Rho GTPases depends on their upstream regulators: the guanine nucleotide exchange factors (GEFs), which promote GTP loading, hence activating the pathway, and GTPase-activating proteins (GAPs), which enhance the enzymatic activity of the GTPase, returning the protein to a GDP-bound state, and, hence, deactivating the pathway. GEFs and GAPs are in turn regulated mainly through cell surface receptors responding to numerous signals (5).

In neurons, the activation state of Rho-family GTPases is affected by brain ischemia *in vivo* or by OGD *in vitro*. In particular, Rac1 has been implicated in OGD/ischemia–induced pathways responsible for delayed neuronal death and cognitive dysfunction (4, 6, 7). Inhibition of ischemia-induced Rac1 activation reduces NADPH oxidase activation and superoxide production in hippocampal CA1 *in vivo*, with consequent reductions in neuronal damage and cognitive impairment (6). We previously demonstrated that Rac1 is activated by OGD in hippocampal neurons but deactivated by the same insult in cortical neurons (4). The Rac GEF Tiam1 interacts with NMDA receptor subunits and is a critical determinant of dendritic spine shrinkage in response to glutamate receptor stimulation during OGD in cultured hippocampal neurons. Furthermore, Tiam1 knockdown by shRNA reduces OGD-induced neuronal death in hippocampal neurons (4). A corresponding GAP to regulate Rac activity in response to OGD remains elusive.

It has recently been demonstrated that the Rac1 GAP BCR functions antagonistically to Tiam1 to tightly regulate Rac1 activity and dendritic spine morphogenesis during neuronal development (8). Activation of Tiam1 is coupled with concurrent deactivation of BCR, resulting in activation of Rac1 and its subsequent downstream effector pathways (8). Here we show that BCR forms a complex with the NMDA receptor subunit NR1 and Tiam1 that is more abundant in hippocampal compared with cortical neurons. OGD causes BCR deactivation and dissociation from the complex in hippocampal neurons but not cortical neurons. Furthermore, BCR knockdown occludes Rac1 activation during OGD in hippocampal but not cortical neurons. Disruption of the BCR–Tiam1 complex with the NR1 binding domain of Tiam1 blocks OGD-induced Tiam1 activation. We propose that the NR1–Tiam1–BCR signaling complex plays a critical role in regulating Rac1 activity and, hence, neuronal vulnerability in response to OGD and that it represents a potential target for therapeutic intervention.

## Results

### BCR is deactivated following OGD in hippocampal but not cortical neurons

**Figure 1.**
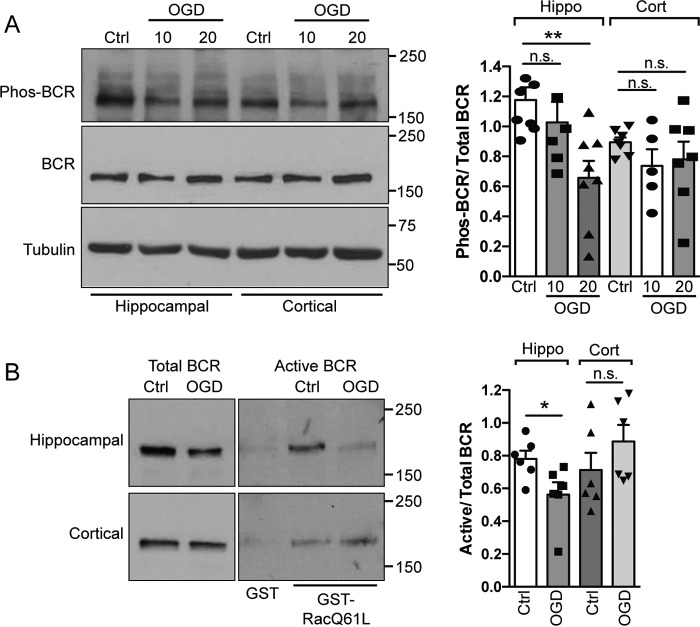
**BCR is deactivated following OGD in hippocampal but not cortical neurons.** *A*, cell lysates from control (*Ctrl*) conditions or after 10 or 20 min of OGD were analyzed by Western blotting with Tyr-177 phospho-BCR antibody. Representative blots show the levels of active (phospho-BCR), total BCR (∼170 kDa), and tubulin in hippocampal (*Hippo*) and cortical (*Cort*) neurons under control and OGD conditions. The graph shows pooled data presented as mean ± S.E. The proportion of active BCR decreased in hippocampal neurons in response to OGD but not in cortical neurons. **, *p* = 0.0072; *n.s.*, not significant (one-way ANOVA, *n* = 6–8 independent cultures). *B*, cell lysates from control conditions or after 20 min of OGD were incubated with GST-Rac1Q61L immobilized on glutathione-agarose beads to isolate active BCR. Cell lysates from control conditions were also incubated with GST as a negative control. Representative blots show the levels of active (Rac1Q61L-bound) and total BCR in hippocampal and cortical neurons under control and OGD conditions. The graph shows pooled data presented as mean ± S.E. The proportion of active BCR decreased in hippocampal neurons in response to OGD. *, *p* = 0.036 (*t* test, *n* = 6 independent cultures).

It has been shown previously that Tiam1 and BCR form a GEF–GAP complex that precisely controls Rac1 activation and thereby modulates dendritic spine development in hippocampal neurons (8). Tightly balanced activation of Tiam1 with concurrent deactivation of BCR results in activation of Rac1 and its subsequent downstream effectors (8). Because Tiam1 is activated by OGD in hippocampal neurons (4), we hypothesized that BCR would exhibit a corresponding change in activation that may contribute to the OGD-induced increase in Rac1 activation observed in hippocampal neurons. To assess BCR activity, we utilized a phospho-specific antibody to Tyr-177 within the BCR N terminus. Phosphorylation of this residue provides a reliable readout of BCR GAP activity and is specifically dephosphorylated upon BCR deactivation (9). We validated the phospho-BCR antibody by knocking down BCR in hippocampal neurons using siRNAs targeted to BCR and Western blotting for both BCR and phospho-BCR (supplemental Fig. S1). These blots showed that the phospho-BCR antibody recognizes a single band at the correct molecular weight, which is markedly reduced by ∼78% upon knockdown with BCR siRNA. We exposed hippocampal and cortical neurons to 10- or 20-min OGD or control conditions and analyzed Tyr-177 phosphorylation by Western blotting. We observed significant Tyr-177 dephosphorylation after 20 min of OGD compared with the control but not after 10 min (Fig. 1*A*). In contrast, there was no change in the phosphorylation state of BCR in cortical neurons after OGD, suggesting that OGD causes reduced BCR activity specifically in hippocampal neurons. We wanted to verify the deactivation of BCR during OGD directly, so we analyzed the proportion of activated BCR using a GAP assay. Following a 20-min OGD insult, hippocampal and cortical neuronal lysates were analyzed using GST pulldown assays using the constitutively active form of Rac1, GST-Rac1Q61L, which is in the GTP-bound state and binds activated GAPs (Fig. 1*B*). In agreement with our phosphorylation results, OGD treatment caused a significant decrease in BCR precipitated with GST-Rac1Q61L, indicating that there is reduced active BCR following OGD in hippocampal neurons. Again, we observed no significant decrease in BCR binding to GST-Rac1Q61L after OGD in cortical neurons, suggesting that this process is specific to hippocampal neurons.

### BCR deactivation during OGD is dependent on NMDARs and Ca^2+^-permeable AMPARs

**Figure 2.**
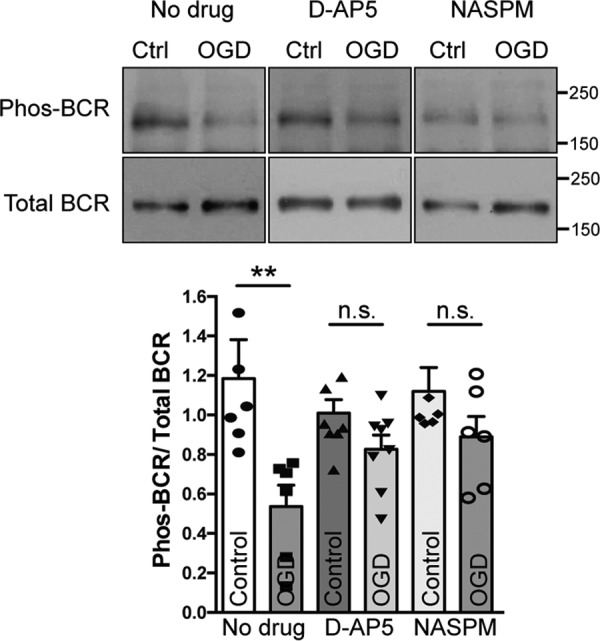
**OGD-induced deactivation of BCR in hippocampal neurons is abolished after blockade of CP-AMPARs or NMDARs.** Cultures were treated with drugs as shown (30 μm NASPM or 50 μm D-AP5), and active BCR was analyzed as described in Fig. 1*A*. Representative blots show the levels of active (phospho-BCR, *Phos-BCR*) and total BCR in hippocampal neurons under control (*Ctrl*) and OGD conditions with or without drug treatments. The graph shows pooled data presented as mean ± S.E., **, *p* = 0.0055; *n.s.*, not significant; *t* test; *n* = 6–8 independent cultures. The OGD-induced deactivation of BCR was abolished by NASPM or D-AP5 treatment.

In hippocampal neurons, OGD causes an increase in Tiam1 and Rac1 activation by a pathway involving NMDARs and Ca^2+^-permeable AMPA receptors (CP-AMPARs), and CP-AMPARs contribute to the excitotoxicity of OGD insult in hippocampal neurons (4). We therefore hypothesized that BCR inactivation might also be dependent on NMDAR and/or CP-AMPAR stimulation. To test this, we applied D-AP5 or NASPM to block NMDARs or CP-AMPARs, respectively, during OGD insult. In the absence of drugs, OGD caused a decrease in BCR phosphorylation in hippocampal neurons, in agreement with the results shown in Fig. 1. Both D-AP5 and NASPM abolished OGD-induced inactivation of BCR, indicating that both NMDAR and CP-AMPAR stimulation are required for this process (Fig. 2).

### The Tiam1–BCR–NR1 complex is more abundant in hippocampal neurons compared with cortical neurons

**Figure 3.**
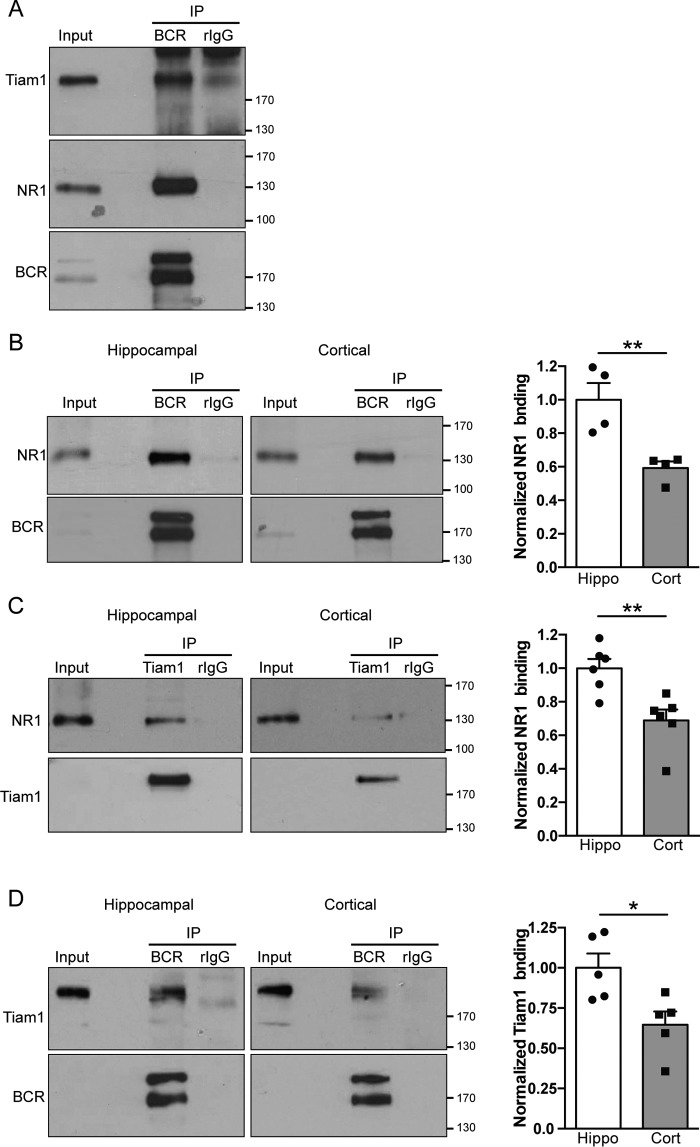
**The BCR–Tiam1–NR1 complex is more abundant in hippocampal neurons than in cortical neurons.** *A*, Western blots of coimmunoprecipitation assays of BCR with Tiam1 and the NMDAR subunit NR1 from cultured hippocampal neurons (*rIgG*, rabbit IgG control). Representative blots show that BCR robustly complexes with Tiam1 and NMDARs. *B*, Western blots of coimmunoprecipitation assays of BCR with NR1 in hippocampal (*Hippo*) and cortical (*Cort*) neurons. The graph shows pooled data presented as mean ± S.E. More BCR associates with NR1 in hippocampal neurons compared with cortical neurons (**, *p* = 0.0087, *t* test, *n* = 4 independent cultures). *C*, Western blots of coimmunoprecipitation assays of Tiam1 with NR1 in hippocampal and cortical neurons. The graph shows pooled data presented as mean ± S.E. More Tiam1 associates with NR1 in hippocampal neurons compared with cortical neurons (**, *p* = 0.0046, *t* test, *n* = 6 independent cultures). *D*, Western blots of coimmunoprecipitation assays of BCR with Tiam1 in hippocampal and cortical neurons. The graph shows pooled data presented as mean ± S.E. More BCR associates with Tiam1 in hippocampal neurons compared with cortical neurons (*, *p* = 0.020, *t* test, *n* = 5 independent cultures).

Tiam1 has been shown previously to interact with NMDARs (10), and BCR interacts with Tiam1 (8), so we predicted that BCR, Tiam1, and NMDARs would form a complex in neurons and that this complex might be central to underlying the downstream signaling effects of OGD in hippocampal neurons. To test this, we performed coimmunoprecipitation (co-IP) experiments using BCR antibodies on lysates prepared from hippocampal neurons. In agreement with our hypothesis, both Tiam1 and NR1 associated with BCR, suggesting that these three proteins are in the same complex and, therefore, ideally placed to mediate downstream signaling in response to OGD (Fig. 3*A*).

We then carried out further co-IP experiments to ask whether cell type–specific differences in the abundance of this complex might underlie the differences in signaling we observed between hippocampal and cortical neurons in response to OGD. Semiquantitative comparisons between hippocampal and cortical neurons of NR1–Tiam1 and NR1–BCR interactions revealed that more Tiam1 and BCR associated with NMDARs in hippocampal neurons compared with cortical neurons (Fig. 3, *B* and *C*). Furthermore, we found that more BCR associated with Tiam1 in hippocampal compared with cortical neurons (Fig. 3*D*). These results indicate that the NR1–Tiam1–BCR complex is more prominent in hippocampal neurons than in cortical neurons and suggest that this complex has a correspondingly greater influence on the regulation of Rac1 activity in hippocampal neurons.

### Dissociation of BCR and Tiam1 following OGD insult

**Figure 4.**
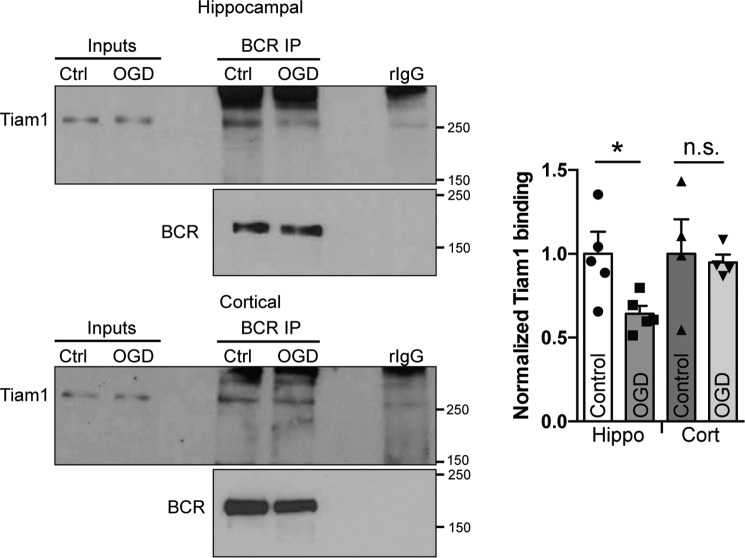
**Dissociation of BCR and Tiam1 following OGD.** Shown are representative Western blots of coimmunoprecipitation assays of BCR, with Tiam1, from cultured hippocampal (*Hippo*) or cortical (*Cort*) neurons treated with control (*Ctrl*) or OGD conditions. The graph shows pooled data presented as mean ± S.E. *, *p* = 0.033; *n.s.*, not significant; *t* test, *n* = 4–5 independent cultures. The Tiam1–BCR complex is disrupted following OGD in hippocampal neurons but not cortical neurons.

OGD causes an increase in Rac1 activation in hippocampal neurons and a decrease in cortical neurons (4). As it has been shown previously that the dissociation of BCR from Tiam1 augments Rac1 activation (8), we investigated the impact of OGD on BCR–Tiam1 interactions in co-IP experiments. Interestingly, OGD caused a decrease in Tiam1 binding to BCR in hippocampal neurons compared with control conditions (Fig. 4). In contrast, there was no change in the Tiam1–BCR interaction after OGD in cortical neurons, suggesting that dissociation of the Tiam1–BCR complex during OGD is specific to hippocampal neurons.

### Higher basal levels of active BCR in hippocampal neurons compared with cortical neurons

**Figure 5.**
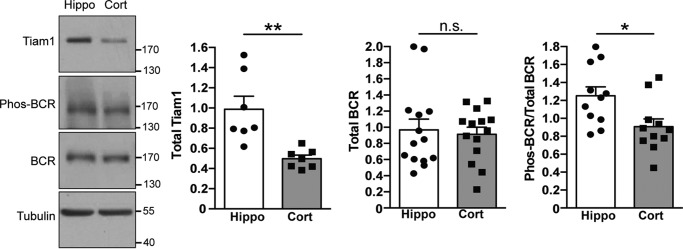
**Higher basal levels of active BCR in hippocampal neurons compared with cortical neurons.** Shown are representative Western blots of hippocampal (*Hippo*) and cortical (*Cort*) lysates probed with antibodies to Tiam1, phospho-BCR, BCR, and tubulin. The graphs shows pooled data presented as mean ± S.E. **, *p* = 0.0033; *, *p* = 0.017; *n.s.*, not significant; *t* test; *n* = 7–14 independent cultures.

**Figure 6.**
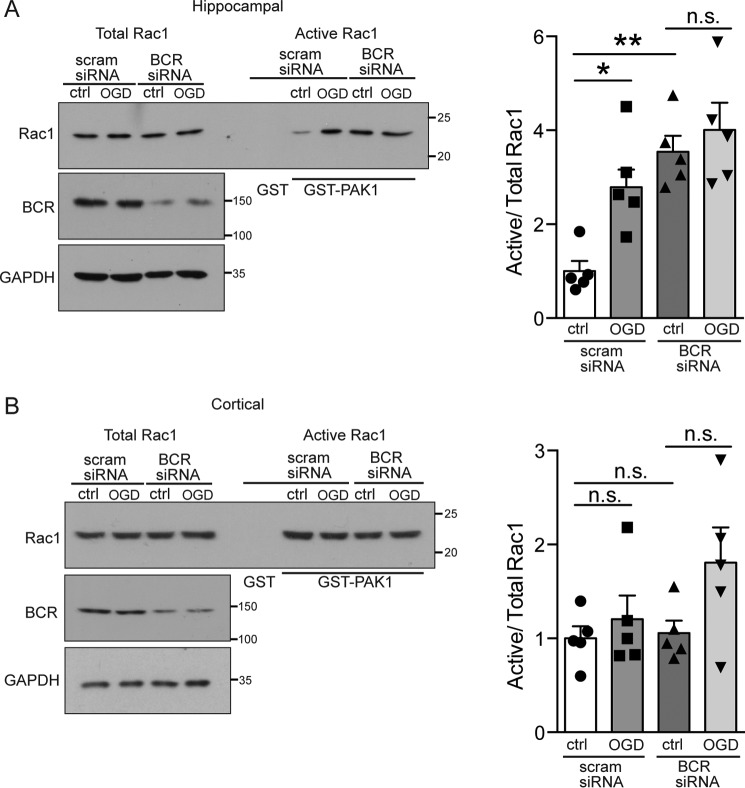
**BCR knockdown occludes OGD-induced increases in Rac1 activity.** *A* and *B*, hippocampal (*A*) or cortical (*B*) lysates treated with scrambled (*scram*) or BCR siRNA from control conditions or after 20 min of OGD were incubated with GST-PAK1 immobilized on glutathione-agarose beads to isolate active Rac1. Cell lysates from control (*ctrl*) conditions were also incubated with GST as a negative control. Representative blots show the levels of active (PAK1-bound) and total Rac1 in hippocampal and cortical neurons under control and OGD conditions. The graph shows pooled data presented as mean ± S.E. *p* = 0.0004; *, *p* = 0.036; **, *p* = 0.0021; *n.s.*, not significant; *n* = 5 independent cultures, one-way ANOVA, Bonferroni post hoc test.

We then asked whether the level of active BCR is higher in hippocampal neurons compared with cortical neurons and is therefore more available for bidirectional modulation by upstream signals. We compared levels of BCR, phospho-BCR, and Tiam1 in lysates from hippocampal and cortical neurons (Fig. 5). As we have shown previously, Tiam1 was expressed at higher levels in hippocampal neurons compared with cortical neurons (4). Interestingly, the levels of total BCR were similar in the two cell types; however, there was a higher proportion of active, phosphorylated BCR in hippocampal neurons compared with cortical neurons (Fig. 5). This suggests that, in hippocampal neurons, there is greater potential for down-regulation of BCR activity compared with cortical neurons, which is consistent with the results presented in Fig. 1. Moreover, basal Rac1 activity, measured by pulldown assays with a GST fusion protein of the Cdc42- and Rac-interactive binding (CRIB) domain of p21-activated protein kinase 1 (GST-PAK1), was significantly increased by siRNA-mediated knockdown of BCR in hippocampal but not cortical neurons (Fig. 6, *A* and *B*), further demonstrating a greater influence of BCR on Rac1 activity in hippocampal neurons.

### BCR knockdown occludes the OGD-induced increase in Rac1 activation

Our data indicate that OGD causes a deactivation of BCR that would contribute to the observed increase in Rac1 activation. To investigate directly a role for BCR in regulating Rac1 activity during OGD, we used GST-PAK pulldown assays in combination with siRNA-mediated knockdown of BCR. The OGD-dependent increase in Rac1 activation in hippocampal neurons was occluded by knockdown of BCR (Fig. 6*A*), indicating that OGD-induced Rac1 activation involves a down-regulation of BCR activity. In contrast, no effect was observed in cortical neurons (Fig. 6*B*).

### The PHCCEx domain blocks Tiam1 interactions and prevents Tiam1 activation during OGD

**Figure 7.**
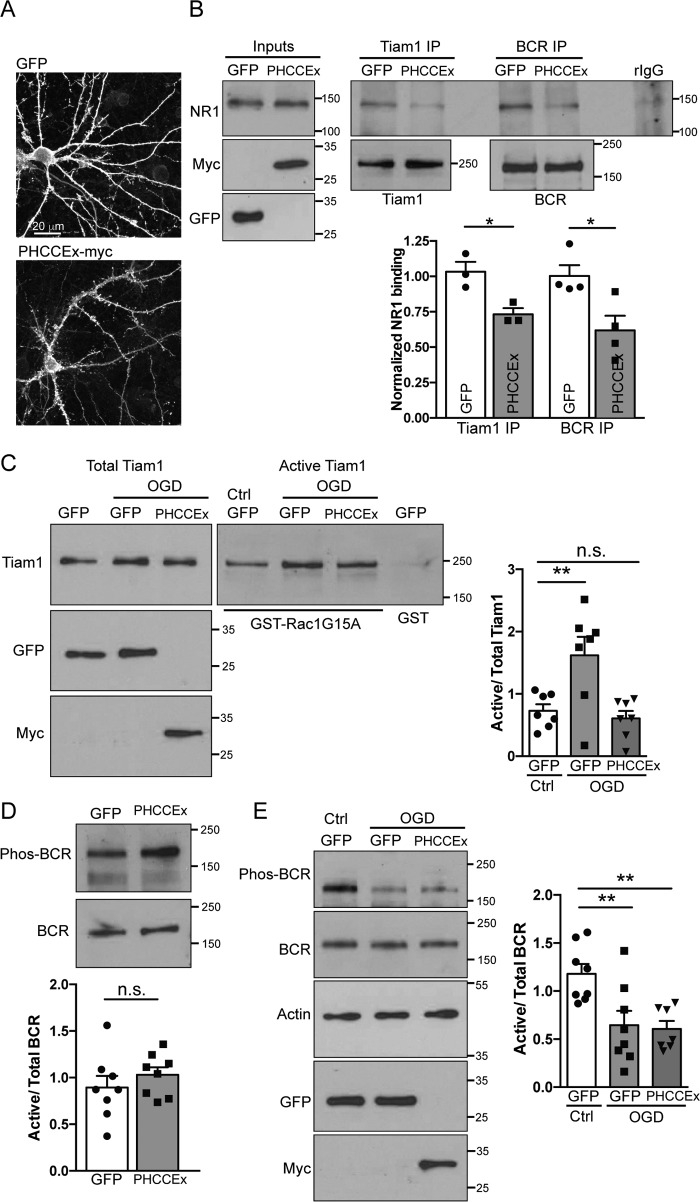
**The PHCCEX domain of Tiam1 blocks Tiam1 activation but not BCR inactivation following OGD.** *A*, confocal images (maximum projections) of hippocampal neurons expressing GFP (GFP fluorescence) or the PHCCEx-myc fragment (anti-myc immunofluorescence). *B*, representative Western blots of coimmunoprecipitation assays of Tiam1 and BCR with NR1 in the presence of GFP or the PHCCEx Tiam1 fragment. Lysates were probed for Myc and GFP antibodies to verify expression of PHCCEx and GFP, respectively. The graph shows pooled data presented as mean ± S.E. Tiam1 IP: *, *p* = 0.025; BCR IP: *, *p* = 0.021; *t* test; *n* = 3–4 independent cultures. Expression of the PHCCEx fragment reduces Tiam1–NR1 and BCR–NR1 interactions. *C*, hippocampal neurons expressing GFP or the PHCCEx Tiam1 fragment were treated under control (*Ctrl*) or OGD conditions for 20 min. Lysates were incubated with GST-Rac1G15A immobilized on glutathione-agarose beads to isolate active Tiam1. Cell lysates from control conditions were also incubated with GST as a negative control. Representative blots show the levels of active (Rac1G15A-bound) and total Tiam1 in hippocampal neurons under control and OGD conditions in neurons expressing GFP or the PHCCEx Tiam1 fragment. Lysates were probed with Myc or GFP antibodies to verify expression. The graph shows pooled data presented as mean ± S.E. The proportion of activated Tiam1 increased in hippocampal neurons in response to OGD, but this was blocked by PHCCEx expression. **, *p* = 0.0085; *n.s.*, not significant (one-way ANOVA, *n* = 7 independent cultures). *D*, expression of the PHCCEx Tiam1 fragment has no effect on BCR phosphorylation. Shown are representative blots of GFP- and PHCCEx-expressing hippocampal neuronal lysates probed with BCR and phospho-BCR antibodies. The graph shows pooled data presented as mean ± S.E. *p* = 0.372, *t* test, *n* = 8 independent cultures. *E*, representative Western blots of lysates from GFP or PHCCEx-expressing hippocampal neurons treated under control or OGD conditions. The graph shows pooled data presented as mean ± S.E. GFP OGD: **, *p* = 0.0072; PHCCEx OGD: **, *p* = 0.0053, one-way ANOVA, *n* = 8 independent cultures. Expression of the PHCCEx Tiam1 fragment has no effect on BCR phosphorylation during OGD insult.

Our results presented here in conjunction with our previous study lead to the hypothesis that uncoupling Tiam1 and BCR from NMDARs might be an effective strategy for inhibiting OGD-induced Rac1 activation in hippocampal neurons. The PHCCEx domain of Tiam1 binds to the NMDAR subunit NR1 (11), so we asked whether expressing the isolated PHCCEx domain would disrupt the NR1–Tiam1 interaction, thereby uncoupling NMDAR activation from downstream Rac1 activation. We virally expressed the myc-tagged PHCCEx domain of Tiam1, or GFP as a control, in hippocampal neurons and found that the protein was expressed at high levels after 12 h (Fig. 7, *A* and *B*). We then analyzed the interactions between NR1 and Tiam1 or BCR in transduced hippocampal neurons. Indeed, the PHCCEx domain reduced the binding of NR1 to both Tiam1 and BCR (Fig. 7*B*).

We then assessed whether PHCCEx domain expression would prevent the OGD-induced changes in Tiam1 and BCR activation in hippocampal neurons. To test the effect of PHCCEx expression on Tiam1 activity, we used a GEF assay to determine the proportion of Tiam1 activated under each condition. Neuronal lysates were analyzed using GST pulldown assays using GST-RacG15A, which is a nucleotide-free mutant of Rac1 and, hence, binds activated GEFs. As we showed previously, Tiam1 activity is increased significantly after 20 min of OGD (4). Expression of the PHCCEx domain completely blocked the OGD-induced increase in Tiam1 activation (Fig. 7*C*), suggesting that an intact Tiam1–BCR–NR1 complex is crucial for Tiam1 activation during OGD. We also assessed the effect of the PHCCEx fragment on BCR inactivation during OGD. PHCCEx domain expression had no effect on the levels of activated BCR (Fig. 7*D*). Interestingly, BCR remained deactivated after OGD in the presence of the PHCCEx fragment (Fig. 7*E*). This suggests that, although NMDAR stimulation is required for OGD-induced BCR deactivation, direct association with NMDARs is not necessary, and other factors are involved in the inactivation of BCR.

## Discussion

In this study, we have shown important mechanistic insights into the signaling pathways that are activated during OGD in hippocampal neurons but not in cortical neurons. Our data show that the Rac1 GAP BCR is deactivated during OGD in hippocampal neurons but not in cortical neurons. BCR interacts with both Tiam1 and NMDARs, and this complex is more abundant in hippocampal neurons compared with cortical neurons. We show that, upon deactivation, BCR is dephosphorylated and dissociates from Tiam1 and NMDARs, a process that is dependent on stimulation of both NMDARs and CP-AMPARs and is specific to hippocampal neurons. Importantly, disrupting the Tiam1–NMDAR–BCR complex can prevent the activation of Tiam1 during OGD.

Although a role for Tiam1 and Rac1 activation during OGD has been described (4), a GAP that deactivates Rac1 during OGD in neurons has not yet been identified. For the first time, we show that a Rac1 GAP, BCR, is involved in the downstream signaling pathways activated by OGD. The deactivation of this GAP during OGD increases the activity of Rac1, which, in the context of OGD, has been shown to be deleterious to neurons, leading to delayed neuronal death and cognitive dysfunction (6, 7). Indeed, inhibition of ischemia-induced Rac1 activation reduces NADPH oxidase activation and superoxide production in hippocampal CA1 *in vivo*, with consequent reductions in neuronal damage and cognitive impairment. Here we show that OGD causes deactivation of BCR, which complements the elevated Tiam1 activity, thereby promoting activation of Rac1. Moreover, knockdown of BCR prevents OGD-induced Rac1 activation in hippocampal neurons, underlining the importance of BCR for controlling Rac1 activity during OGD in these neurons. This strongly suggests that deactivation of BCR is an important factor underlying the increased Rac1 activation during ischemia, which contributes to neuronal damage.

Our results show that there are higher levels of active BCR under basal conditions in hippocampal neurons compared with cortical neurons. This mirrors the higher expression of Tiam1 in hippocampal neurons and suggests that higher levels of active BCR are required to counteract Tiam1 and, therefore, provide dynamic modulation of Rac1 activity. The deactivation of BCR upon OGD insult is dependent on both NMDAR stimulation and activation of CP-AMPARs, which is the same for Tiam1 activation during OGD (4). Because BCR, Tiam1, and NMDAR form a physical complex that is modulated during OGD, this likely places BCR in the same signaling pathway as Tiam1 to modulate Rac1 activity in response to external signals mediated by NMDARs and CP-AMPARs. Importantly, deactivation of BCR occurs in the same timeframe as Tiam1 activation (within 20 min of OGD), supporting the idea that this complex is dynamically controlling Rac1 activation in response to OGD. We show that the inactivation of BCR during OGD is dependent on the activation of CP-AMPARs, which is also the case for Tiam1 activation (4). During OGD, CP-AMPARs are trafficked to synapses in an NMDAR-dependent manner, and this expression of CP-AMPARs is required for delayed neuronal cell death (12). Hippocampus-specific modulation of BCR during OGD is therefore an additional component of a larger process that likely makes this brain region more susceptible to delayed cell death compared with the cortex.

Rac1 and its regulators play a central role in the remodeling of dendritic spines and controlling synaptic plasticity (13, 14). Accordingly, BCR has been shown to be important for synapse formation, synaptic plasticity, and learning and memory by negatively regulating spine development (8, 15). Moreover, an appropriate balance of the activities of Tiam1 and BCR is necessary for proper spine morphogenesis during development (8). Dendritic spine structure and function are disrupted in multiple neuropsychiatric and neurodegenerative disorders (16), and dendritic spines are altered following ischemia *in vivo* and OGD *in vitro* (4, 17–20). We have shown previously that Tiam1, a key regulator of spine shape and size, is activated during OGD and is likely to be responsible for the maintenance of spine size in hippocampal neurons during OGD (4). Given the role of BCR at synapses, it is therefore plausible that BCR deactivation could restrict dendritic spine shrinkage during OGD, thereby maintaining excitatory synaptic inputs, promoting excitotoxicity, and contributing to delayed cell death.

The GAP activity of BCR is enhanced by phosphorylation at Tyr-177. In neurons, this site is phosphorylated by Fyn kinase and dephosphorylated by the phosphatase PTPRT; therefore, Fyn/PTPRT are thought to control BCR GAP activity (9). Moreover, it has been suggested that Fyn has a dual function in this pathway because phosphorylation of PTPRT by Fyn reduces its phosphatase activity, further promoting BCR activation (9, 21). Although little is known about the regulation or pathophysiological role of PTPRT, it has been shown to be involved in the regulation of synapse development and neuronal morphology (21, 22). Fyn associates with NMDARs, and its activity is implicated in the pathogenesis of brain ischemia (23, 24); hence, both proteins are likely candidates for mediating the NMDAR-dependent deactivation of BCR in response to OGD. Further work will determine the role of these signaling proteins in modulating BCR function in the context of OGD/ischemia.

Our experiments indicate that expressing the isolated PHCCEx domain of Tiam1 disrupts the NR1–Tiam1–BCR complex. Although the structure of this domain has been described in detail (11), and it has been shown to alter spine morphology (8), this is the first demonstration of its use as a dominant-negative interfering peptide to disrupt the NR1–Tiam1–BCR complex. This intervention blocks OGD-induced Tiam1 activation but not BCR deactivation, demonstrating that Tiam1 activation following OGD is dependent on the integrity of its interaction with NMDARs. These findings also suggest that OGD-induced BCR deactivation does not require physical association of BCR with NMDARs/Tiam1, despite our observation that NMDAR activation is required for reducing BCR activity. We also show that, although OGD causes both BCR deactivation and disruption of BCR interactions with NR1 and Tiam1, disruption of the complex does not in itself cause a reduction in BCR activity. Alternatively, deactivation of BCR might be upstream of dissociation from the NR1–Tiam1 complex so that dephosphorylated BCR has a reduced affinity for its binding partners. Because down-regulating Tiam1 in hippocampal neurons protects hippocampal neurons from OGD-induced cell death, our results further suggest that the PHCCEx domain or similar disrupting peptide might prove to be a useful tool to disrupt aberrant signaling caused by ischemia in specific brain regions and may therefore be neuroprotective.

## Experimental procedures

### Primary neuronal cell culture

Rat embryonic hippocampal and cortical neurons were prepared by dissection of embryonic day 18 Wistar rat embryos of either sex using standard procedures. All procedures were approved by and performed in accordance with guidelines of the Animals (Scientific Procedures) Act 1986 and the University of Bristol policy on working with animals. Neurons were plated on poly-l-lysine–coated 3.5-cm plastic dishes at 400,000 cells/dish and cultured in Neurobasal medium (Gibco) supplemented with B27 (Gibco) and 2 mm Glutamax. Primary cell cultures were used for experiments at 16 to 20 days *in vitro*.

### Transfection of siRNAs

Hippocampal and cortical neurons were transfected with siScrambled (Dharmacon ON-TARGETplus Non-targeting Pool, D-001810-10) and siBCR (Dharmacon SMARTpool, M-094573-01) using Lipofectamine RNAimax (Invitrogen) according to the instructions of the manufacturer. Briefly, 10 μl of RNAimax and 2 μl of siRNA (10 μm stock) were each diluted in 150 μl of plain Neurobasal medium and then combined to allow siRNA–lipid complexes to form. 10 min post-incubation, the complexes were added dropwise to a dish of 11 days *in vitro* neurons plated on 3.5-cm dishes. 72 h post-transfection, the neurons were harvested for Western blotting or used for OGD experiments.

### Antibodies

The following antibodies were used: polyclonal anti-BCR (Santa Cruz Biotechnology, sc-886, 1:1000), polyclonal anti-phospho-BCR Tyr-177 (Cell Signaling Technology, 3901, 1:1000), polyclonal anti-Tiam1 (IP, Santa Cruz Biotechnology, sc-872; Western blot, Bethyl, A300-099A, 1:1000), monoclonal anti-tubulin (Sigma, 1:5000), monoclonal anti-Rac1 (BD Biosciences, 610650, 1:1000), monoclonal anti-NR1 (Millipore, MAB1586, 1:500), monoclonal anti-Myc (Santa Cruz Biotechnology, sc-40, 1:400), and monoclonal anti-GFP (Neuromab, 75-131, 1:400).

### OGD

Cell cultures were washed three times with HEPES-buffered saline (25 mm HEPES (pH 7.5), 137 mm NaCl, 5 mm KCl, 1.5 mm CaCl_2_, and 1.5 mm MgCl_2_, containing 15 mm sucrose for the OGD condition or 15 mm glucose for the control condition). OGD cultures were incubated in a hypoxic chamber (MACS-VA500 microaerophilic work station, Don Whitley Scientific) at 37 °C, 95% N_2_, and 5% CO_2_. Control cultures were incubated at 37 °C, 5% CO_2_ for the same time period as for OGD. After OGD, the cell cultures were lysed immediately in lysis buffer (25 mm HEPES, 150 mm NaCl, 0.5% Triton X-100, 1% protease inhibitor mixture (Roche), phosphatase inhibitor mixture 3 (Sigma), 10 mm NaF, and 1 mm Na_3_VO_4_) and were either analyzed by SDS-PAGE or used for subsequent biochemical assays.

### GST pulldown assays

For active Tiam1, BCR, and Rac1 GST pulldowns, GST-Rac1G15A, GST-Rac1Q61L, and GST-PAK were expressed and purified from BL21 bacterial cultures. 1 liter of bacterial culture was induced for 2 h using 0.2 mm isopropyl 1-thio-β-d-galactopyranoside at 30 °C. The bacteria were then pelleted and resuspended in 50 ml of HTG buffer (25 mm HEPES, 150 mm NaCl, 1% Triton X-100, and 10% glycerol (pH 7.5)). Bacteria were sonicated on ice, and debris was removed by centrifugation. GST fusion proteins were immobilized on glutathione-agarose beads (Sigma) in HTG buffer at 4 °C for 1 h. Beads were incubated with hippocampal or cortical lysate for 1 h at 4 °C in lysis buffer. After washing the beads with the same buffer, protein levels were detected by Western blotting using antibodies to either Tiam1, BCR, or Rac1 (Bethyl, Santa Cruz Biotechnology, and BD Biosciences, respectively). GST bound to glutathione-agarose beads was used as a negative control. Bound proteins were detected by Western blotting as described in Ref. 25.

### Semiquantification of Western blots

Western blot films were scanned and analyzed using ImageJ software (National Institutes of Health) by creating a rectangle around the bands to be analyzed and producing intensity plots from which the intensity of each band could be determined. For BCR phosphorylation analysis, phospho-BCR bands were normalized to total BCR bands from the same blot. For GST pulldown and immunoprecipitation, the bound protein bounds were normalized to their respective input bands. To analyze the protein levels of Tiam1 and BCR, the bands were normalized to loading controls (tubulin and GAPDH). Error bars indicate standard error, and statistical tests were performed using GraphPad Prism.

### Drug treatments

1-Naphthylacetyl spermine (NASPM, 30 μm, Tocris, Minneapolis, MN) and d-(−)-2-amino-5-phosphonopentanoic acid (D-AP5, 50 μm, Tocris) were used to block CP-AMPARs or NMDARs, respectively. Oxygen/glucose deprivation was performed in the presence of either drug (in the case of D-AP5, primary cultures were preincubated with 50 μm of the drug for 5 min before OGD and for the duration of OGD). Rac1 or Tiam1 GST pulldowns were performed immediately after OGD.

### Coimmunoprecipitation experiments

Coimmunoprecipitation experiments from cultured neurons were performed by lysing cultured neurons in pulldown buffer (50 mm Tris (pH 7.5), 1% Triton X-100, 150 mm NaCl, 1 mm EDTA, 1% protease inhibitor mixture, phosphatase inhibitor mixture 3 (Sigma), 10 mm NaF, and 1 mm Na_3_VO_4_) and solubilized for 30 min. Solubilized material was ultracentrifuged at 16,000 × *g* for 40 min at 4 °C, and the supernatant (solubilized protein) was incubated with 2 μg of antibody for 2 h at 4 °C. To precipitate complexes, 15 μl of protein G beads were added for 30 min at 4 °C. Beads were then washed extensively, and bound complexes were analyzed by SDS-PAGE and Western blotting.

### Production of the Tiam1 PHCCEx domain

Sindbis virus expressing the Tiam1 PHCCEx domain with an additional myc tag was produced by amplification of the Tiam1 PHCCEx domain (11) and subsequent cloning into the pSinRep5 plasmid, followed by Ambion virus production tools. 30 μl of virus were added to 3.5-cm dishes of hippocampal neurons and allowed to express for 12 h. Infected cells were then processed as described above by GST pulldown or coimmunoprecipitation.

### Confocal microscopy

Hippocampal neurons were grown on glass coverslips for microscopy and infected with virus as described above. 12 h post-infection, neurons were fixed in 4% paraformaldehyde (Thermo Fisher) in PBS (Sigma) supplemented with 2% sucrose at room temperature. Cells were blocked in 0.1% Triton X-100/3% BSA (Sigma) in PBS for 1 h and incubated with either anti-GFP or anti-myc antibodies (both at 1:400, Neuromab) for 1 h at room temperature. Cells were then incubated in goat anti-mouse Alexa Fluor 488 for 45 min (1:1000, Thermo Fisher) and mounted onto slides with Prolong Gold Antifade reagent (Thermo Fisher). Coverslips were imaged with a Leica SP5 confocal system under a ×63/1.4 numerical aperture oil immersion objective using filters set up to image GFP. Leica software was used to acquire z-stacks with 0.37-μm step size, which are displayed as maximum projections produced in ImageJ.

## Author contributions

J. G. H. conceived the study. K. R. S. and J. G. H. designed the experiments, supervised the project, and wrote the manuscript. K. R. S. performed and analyzed the experiments. D. R. performed experiments.

## Supplementary Material

Supplemental Data

## Footnotes

3 The abbreviations used are:

OGD: oxygen and glucose deprivation
GEF: guanine nucleotide exchange factor
GAP: GTPase-activating protein
CP: Ca^2+^-permeable
AMPAR: AMPA receptor
NMDAR: NMDA receptor
NASPM: 1-naphthylacetyl spermine
D-AP5: d-(−)-2-amino-5-phosphonopentanoic acid
IP: immunoprecipitation
ANOVA: analysis of variance
BCR: breakpoint cluster region
PHCCEx: pleckstrin homology, coiled coil and extra
PTPRT: receptor-type tyrosine-protein phosphatase T.
