# Supplementary material for: Differential regulation of the Rac1 GTPase–activating protein (GAP) BCR during oxygen/glucose deprivation in hippocampal and cortical neurons
Source: J Biol Chem. 2017 Oct 18;292(49):20173–83. doi: 10.1074/jbc.M117.796292 (PMC5724004; doi:10.1074/jbc.M117.796292)
Supplement: Supplemental Data [file supp_292_49_20173__index.html]

Differential regulation of the Rac1 GTPase–activating protein (GAP) BCR during oxygen/glucose deprivation in hippocampal and cortical neurons — Regulation of BCR during OGD in hippocampal neurons — Supplemental Data 

# Differential regulation of the Rac1 GTPase–activating protein (GAP) BCR during oxygen/glucose deprivation in hippocampal and cortical neurons

## Supplemental Data

- Figure S1 (.jpg, 560 KB) - BCR siRNA characterisation
