# Supplementary figures and images for: Differential regulation of the Rac1 GTPase–activating protein (GAP) BCR during oxygen/glucose deprivation in hippocampal and cortical neurons
Source: J Biol Chem. 2017 Oct 18;292(49):20173–83. doi: 10.1074/jbc.M117.796292 (PMC5724004; doi:10.1074/jbc.M117.796292)

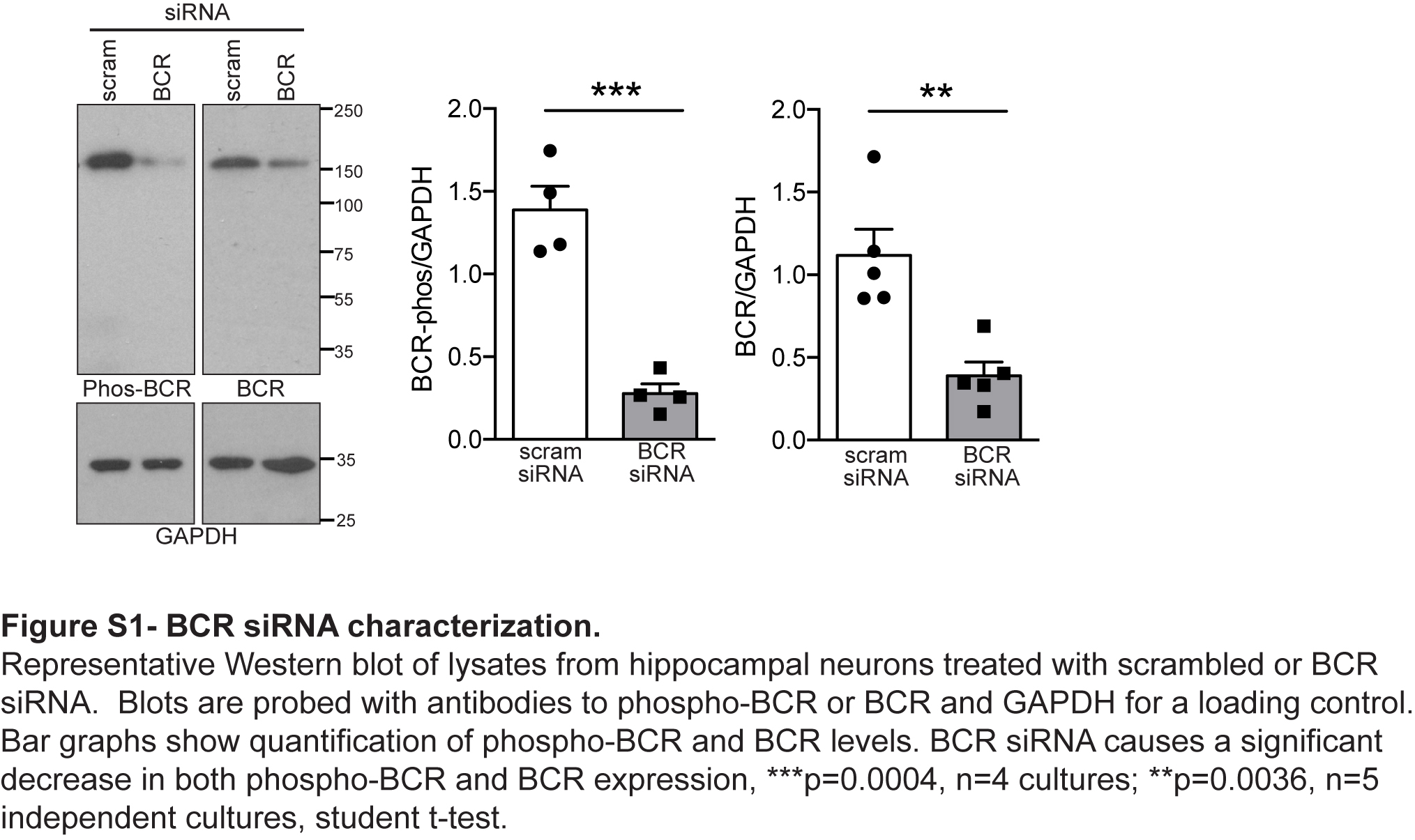

Supplement: Supplemental Data [file 10.1074_M117.796292_jbc.M117.796292-1.jpg]
